# Caregiver Perceptions of Autism and Neurodevelopmental Disabilities in New Delhi, India

**DOI:** 10.3390/ijerph20075291

**Published:** 2023-03-28

**Authors:** Georgia Lockwood Estrin, Supriya Bhavnani, Rashi Arora, Sheffali Gulati, Gauri Divan

**Affiliations:** 1 School of Psychology, University of East London, Arthur Edwards Building, Water Lane, London E15 4LZ, UK; 2 Child Development Group, Sangath, Bardez 403501, India; supriya.bhavnani@sangath.in (S.B.); gauri.divan@sangath.in (G.D.); 3 Center of Excellence and Advanced Research on Childhood Neurodevelopmental Disorders, Child Neurology Division, Department of Pediatrics, All India Institute of Medical Sciences, New Delhi 110029, India; sheffaligulati@gmail.com

**Keywords:** autism, neurodevelopment disability, caregiver perspectives, low- and middle-income countries

## Abstract

Evidence suggests that parenting an autistic child or a child with neurodevelopmental disabilities can be more challenging than parenting a child meeting their developmental milestones, especially when there is a dearth of support services, such as in low- and middle-income countries (LMICs). Despite the majority of the world’s children residing in LMICs, there are limited studies examining the understanding of developmental disorders and autism in these regions. We therefore aim to investigate perceptions of autism and developmental disabilities in caregivers of children in an urban setting in New Delhi, India. Thirteen semi-structured interviews with parents/caregivers of children were conducted in three groups: (1) caregivers with a child with a diagnosis of autism spectrum disorder (ASD); (2) caregivers with a child with a diagnosis of intellectual disability (ID); (3) and caregivers with children meeting their developmental milestones. Transcripts were analysed using framework analysis. Three themes on the impact of cultural and contextual factors on the recognition, interpretation, and reporting of autistic symptoms are discussed, and additional themes focus on the impact of diagnosis and family support. Our findings highlighted a vital need for greater community awareness and recognition of autism in India, for example through community and healthcare training, which may help to reduce stigma and facilitate wider family support.

## 1. Introduction

Autism spectrum disorder (ASD; also referred to as “autism”) is a complex disorder and has been defined, according to DSM-V, by difficulties in social communication and interaction, as well as restricted interests and increased repetitive behaviours [1]. Research suggests that parenting an autistic child can be more challenging than parenting a child meeting their developmental milestones, especially due to the dearth of support services available [2]. This is particularly the case in low-resource settings, such as in low- and middle-income countries (LMICs) [3,4].

It has been estimated that there are over 2 million autistic children living in India, accounting for over a quarter of the global prevalence [5,6]. However, despite this, the overwhelming majority of our understanding about autism relies on research conducted in Western, high-income countries (HICs) [7,8]. We have relatively limited evidence from countries where a majority of autistic children reside. This is important because differences in sociocultural norms between contexts can significantly impact the process of identifying symptoms and help-seeking, diagnosis and health services for autism and developmental disorders across the globe [8,9,10]. Moreover, low awareness about autism in the local community has been associated with increased levels of stigma and parental stress, as well as reduced support for families, which in turn may increase the likelihood of a delay in diagnosis and help-seeking [2,11]. A greater understanding of the experience of families of autistic children from LMICs is therefore fundamental to the development of improved access to healthcare in underserved populations.

Research into the experience of autism from parental perspectives has commonly focused on parental stress and their associated outcomes. Theoretical models for stress in families of children with ASD or developmental disabilities have focused on life stressors, child characteristics, family and individual resources and support [12]. More recently, a recent scoping review of autism research conducted in India identified that a diagnosis of ASD generates apprehension, worry, and anxiety in parents [13]. This review located a total of 18 articles which investigated parental perceived burden and help-seeking attitudes, professional perspectives and available service provision in the community; however, this review called for the need of additional research focusing on family perspectives. There is also evidence indicating that parents of children with an ASD diagnosis report higher levels of anxiety, depression, and stress, and lower levels of well-being when compared to parents of children meeting their developmental milestones, and parents of children with developmental disabilities [2].

This paper aims to expand current literature and examine parental perceptions of autism in an urban population in New Delhi, India. We do this by exploring caregivers’ perceptions of autism and developmental disorders across three groups: parents of children with a diagnosis of ASD, parents of children diagnosed with another neurodevelopmental condition (specifically a diagnosis of intellectual disability (ID)) but not autism, and parents of children meeting their developmental milestones. This approach allows for an in-depth investigation of perceptions of autism from parents with and without direct experience of autism. We employ a novel conceptual framework that has been developed by de Leeuw and colleagues to enhance and guide our understanding of the cultural and contextual factors on autism [9]. The framework considers the interrelated identification and diagnostic process at four levels: expression, recognition, interpretation, and reporting of autism symptoms. In this paper we aim to utilize this framework at three levels (recognition, interpretation, and reporting of autism symptoms) to explore the contextual and cultural factors impacting the recognition, interpretation and reporting of autism in an urban setting in Delhi, India.

Increasing our understanding of perceptions of autism and the impact of a child’s autism diagnosis across different cultures and settings is important to strengthen systems of care to increase awareness [14], reduce stigma and increase the support to children and their families through an improved update of services [15].

## 2. Materials and Methods

### 2.1. Participants

This qualitative study was conducted with a sub-sample of caregivers of children who were participating in a larger study in New Delhi, India [16]. All caregivers interviewed were mothers. Participants from the larger study were recruited into one of three groups: children aged 3–5 years with an ASD diagnosis, a diagnosis of ID, or children meeting their developmental milestones. Samples were matched by age and gender, and full details of this sample and recruitment strategy can be found in [16].

Participating families in the clinical groups were recruited from a government-run tertiary care centre: the Department of Paediatric Neurology at All India Institute of Medical Sciences (AIIMS). All children in the clinical groups had a clinical diagnosis based on DSM-V [1] criteria from a senior paediatrician at this hospital. Children in the comparison group (children meeting their developmental milestones) were recruited from communities in the New Delhi region, through contacts established by the research team’s existing child development studies.

### 2.2. Study Procedures

As part of the larger study [16], questionnaires relating to family demographics were administered verbally with the parent/caregiver (as detailed in [17]), and developmental measures and autism traits were assessed in all children by a local psychologist who was part of the research team. The Developmental Profile 3 (DP-3) [18] was used to assess child development. It is routinely used in clinical practice and has been extensively used for research purposes in this setting [16,19,20,21]. As outlined in [16], for this study, the DP-3 was administered by an experienced psychologist on the research team, and it included both parental report and the assessor’s observation of child behaviour. Autism traits were assessed using the AIIMS-Modified-INDT-ASD [5,19,22], which is a tool developed and validated for an Indian paediatric population for clinical assessment of ASD [23].

Informed consent for the qualitative study was obtained prior to the start of the interview. For this sub-study, we aimed to recruit 10–15% of study participants from the larger study [16]. Thirteen semi-structured in-depth interviews were conducted with caregivers in Hindi. Interviews took place in a private space within a community centre or within a healthcare facility, and where interviewer and interviewee were able to speak without interruption. The interviews were conducted by a fluently bilingual (Hindi, English) Indian female research assistant (co-author RA), with master’s level qualification in psychology.

Interview topic guides were developed for interviews with caregivers. A stepped approach was taken to explore caregivers’ understanding of autism, developmental disorders and child development in general. All caregivers were first asked if they had heard of, and what they understood by the term “autism spectrum disorders”, and if caregivers had not heard of this term, they were asked ‘what do you understand of the term developmental disorders?”. If they had not heard of this term, caregivers were asked what they understood by “child development”. The topic guide was then used to explore the caregivers’ understanding, as well as their experiences, of obtaining a diagnosis and access to healthcare, and the impact of a diagnosis for the child and family. If the parent had not heard of “developmental disorders” (in the group of parents with a child meeting their developmental milestones), the interviewer probed to find out if the participant was aware of children in their communities “who were not developing in the same way as other children”. All participants were able to consider a child with developmental delays, and corresponding questions from the interview focused on such children. For parents of children meeting their developmental milestones, parents were asked to speculate on these topics to get a sense of a community-level perception. All interviewees consented to recording on a voice recorder. Interviews were then translated and transcribed in full to English by an experienced bilingual researcher. Transcriptions were also checked for accuracy by a senior bilingual researcher on our research team.

### 2.3. Ethical Approval

All experimental procedures were reviewed and approved by the Institutional Ethics Committees of Sangath (approval number: GD_2018_39) and the Department of Psychological Sciences, Birkbeck, University of London (reference number 171897), and the AIIMS Institutional Ethics Committee (approval number RP-37/2018, RP-49/2019).

### 2.4. Analysis

Full transcripts from the in-depth interviews were analysed using framework analysis. This approach was taken due to it being widely used in healthcare research [11,24]. Our coding framework was theoretically driven and based on Leeuw et al.’s [9] conceptual framework. We focused on their three levels of cultural and contextual factors: recognition, interpretation and reporting of autism symptoms. For the analysis, we followed the five steps of framework analysis [25]. Familiarization involved first immersion in interview transcripts to draw out key themes in the data. The framework was then built, which included a combination of concepts from the de Leeuw et al. [9] framework, as well as emergent themes [25] relating to the perceived impact of an autism diagnosis. GLE then independently indexed the interview data by applying it to the framework. SB reviewed this indexing, and themes were ordered and refined systematically through discussion between GLE and SB. GLE and SB then explored patterns within and across themes during the final stage of data analysis.

## 3. Results

**Figure 1:**
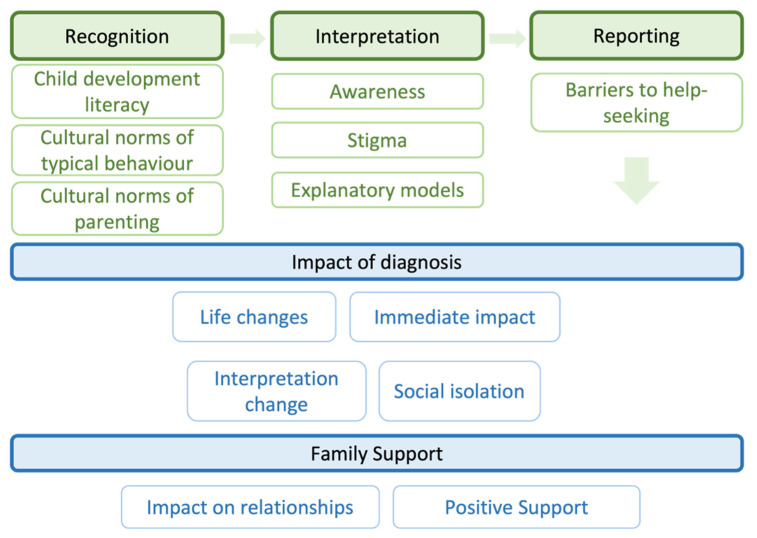
Overall Themes from the analysis.

We reported our findings from the analysis as five themes. We first reported on three key themes from the de Leeuw et al. [9] framework on the impact of culture on the recognition, interpretation, and reporting of autistic symptoms. The two additional themes were: the impact of diagnosis, and family support (Figure 1). The demographics of participants can be found in Table 1.

### 3.1. Recognition

Based on the de Leeuw et al. [9] conceptual framework, our analysis identified the following sub-themes under the theme of “Recognition of symptoms”: (1) child development literacy; (2) cultural norms of typical behaviour; and (3) cultural norms of parenting.

#### 3.1.1. Child Development Literacy

Our results illustrated a striking lack of awareness in the community about developmental milestones for child development and autism.

In the comparison group, caregivers described the community as being “unaware” or considering autism traits as “normal”.

“*Parents are either uneducated or are unaware… They won’t be knowing that these things [autism, developmental delay] exist and so how would they know what is happening [with their child].*”.

(mother of a child meeting their developmental milestones)

“*They [parents] would have realized late that our child is behaving in an unusual way. Else… [thinks]. This could be the only reason. Might be, they don’t pay much attention to the child. They would be thinking “it is normal.*”.

(mother of a child meeting their developmental milestones)

Caregivers in the clinical groups stated that they knew something was ‘different’, but had not heard of autism:

“*We have seen this problem for the first time … we never heard that children have such problem.*”.

(mother of a child with an ASD diagnosis)

#### 3.1.2. Cultural Norms of Typical Behaviour

In the comparison group, caregivers described children with developmental disorders as being “like children who grow physically but not mentally,” (mother of a child meeting their developmental milestones).

“*Such children are not like the normal children. They don’t do things as those are meant to be done.*”.

(mother of a child meeting their developmental milestones)

In the clinical groups, caregivers described that they felt that their child was like other children, and this made it difficult to know there were symptoms of autism/developmental disability.

“*No one can say by looking at him that he might have any problem*.”.

(mother of a child with an ASD diagnosis)

Delayed or lack of speech was mentioned by caregivers as the key in understanding that there was a problem, and so in families where the child was progressing with language development, this made it more difficult to recognize that other difficulties may be present.

“*Like other normal children, he is learning to speak. He is like a normal child*.”.

(child at 6 years, mother of a child with an ID diagnosis)

#### 3.1.3. Cultural Norms of Parenting

Caregivers in the clinical groups highlighted how important it was for them to have assistance from the wider family, school, as well as experienced childcare support, such as from an employed child-carer or extended family members, e.g., grandparents, in helping to recognise their child’s difficulties. This highlighted the collective approach to raising a child in these communities.

“*I talked to my housemaid in [name of the hometown state]. She told me to consult the doctor otherwise the problem will progress… I talked to her first….*”.

(mother of a child with an ID diagnosis)

### 3.2. Interpretation

Based on the de Leeuw et al. [9] conceptual framework, our analysis identified the following sub-themes under the “Interpretation of symptoms”: (1) awareness; (2) explanatory models; and (3) stigma.

#### 3.2.1. Awareness

In the comparison group, caregivers talk about children with a developmental disorder and autism as being like a “young child.”

“*His brain is like that of a young child… he would be 27–28 years old. He behaves like a child*.”.

(mother of a child meeting their developmental milestones)

Caregivers of a child with an ASD diagnosis spoke about how it was easy to ignore symptoms, as they were similar to other children in some ways, and no one considered autism as a possible explanation for their concerns.

“*No one thought that it could be an autism symptom*.”.

(mother of child with ASD diagnosis)

“*Normal kids can’t also do everything. Every kid has different abilities*.”.

(mother of a child with an ASD diagnosis)

#### 3.2.2. Explanatory Models

Different explanatory models for symptoms were described in the ASD compared to the comparison groups. Caregivers of children with a child meeting their developmental milestones focused explanatory models on nutrition, bad spirits or lack of care given to children with developmental disorders.

“*If a pregnant mother doesn’t eat specific medicine or dose [e.g., iron tablets etc], it leads to such or any other problems in child*.”.

(mother of a child meeting their developmental milestones)

“*Society thinks they give birth to the child but are not taking care of them*.”.

(mother of a child meeting their developmental milestones)

In the ID group, caregivers focused on biological explanations for their child’s ID diagnosis, and whilst the biological reasons were not necessarily understood, there was a general consideration of “weakness”, rather than social aspects causing their child’s symptoms.

“*[The child] is unable to develop due to some weakness.*”.

(mother of a child with an ID diagnosis)

Comparatively, caregivers of children with an ASD diagnosis focused on social explanations for their child’s autism. This included describing a lack of optimal environment in the family as an explanation for autism symptoms.

“*The environment of the house also makes a difference. Joint family produces more growth, nuclear less*.”.

(mother of a child with an ASD diagnosis)

We found that mothers of children with an ASD diagnosis expressed feelings of self-blame or perceived that other members of the family blamed them for their child’s autism, for example, for a lack of attention given to the child, or difficulties experienced during pregnancy.

“*I also didn’t pay regular attention. We can’t be with the child every time—sometimes when we are busy with our work,… It happens by being alone*.”.

(mother of a child with an ASD diagnosis)

“*I was very stressed during pregnancy so my maternal aunt usually says, ‘You took stress during pregnancy which has affected the child.*”.

(mother of a child with an ASD diagnosis)

#### 3.2.3. Stigma

Interestingly, only in the ASD group did caregivers discuss their experience of stigma and discrimination. This manifested in a feeling of blame of the mother for not “caring” or being too “proud”, or for something the mother did in pregnancy that “caused” their child’s difficulties. Caregivers of children with ID diagnoses did not describe these as a significant experience, even when probed.

“*Some says, ‘you were proud [arrogant] of giving birth to an adorable boy [and so this happened]’.*”.

(mother of a child with an ASD diagnosis)

### 3.3. Reporting

Based on the de Leeuw et al. [9] conceptual framework, our analysis identified barriers to help-seeking perceived by caregivers.

Barriers to Help-Seeking

For caregivers in the comparison groups, caregivers reported mainly financial or logistical barriers to reporting symptoms.

“*Some don’t have money or some don’t have time. It could be either of them.*”.

(mother of a child meeting their developmental milestones)

“*It would be financial problem, what else… One can’t leave the child just like that. They will go. They will try. But if one doesn’t have money then till where they can go.*”.

(mother of a child meeting their developmental milestones)

“*There could be some problem at home or we don’t get the reservation [for tickets]… We might be late if have any family problem otherwise we visit on the scheduled date of appointment.*”.

(mother of a child with an ID diagnosis)

However, in the ASD group, a more complex mixture of barriers were identified, including maternal lack of agency, financial difficulties, stigma, and a disbelief that symptoms were those of autism.

“*It seemed to me that there was some problem. But no one in the society listens to us ladies, no matters how much educated we are (sic).*”.

(mother of a child with an ASD diagnosis)

“*I knew if I disclose it [child’s diagnosis]… then no one will allow my child and me to come in.*”.

(mother of a child with an ASD diagnosis)

### 3.4. Impact of Diagnosis

Further themes were identified, which focused on the impact of a diagnosis on the family, and these included: (1) immediate impact; (2) social isolation; (3) child needing more care and attention; (4) life changes; (5) change in interpretation of developmental disorder.

#### 3.4.1. Immediate Impact of Diagnosis

The immediate impact of an ASD diagnosis for caregivers included feelings of depression, concern for the future, and disbelief.

“*I didn’t make food for three days [when hearing of diagnosis] [Mother felt overwhelmed and almost cried]. He was young that time. I didn’t do anything except lying on bed and crying*.”.

(mother of a child with an ASD diagnosis)

Comparatively, in the ID group, caregivers spoke of worry and being upset that their child is suffering, but did not speak of stigma, discrimination or feelings isolation, as expressed by mothers with a child diagnosed with ASD.

“*It hurts you deeply that your child is suffering so much. You feel sad*.”.

(mother of a child with an ID diagnosis)

#### 3.4.2. Social Isolation

Social isolation was discussed by caregivers in the ASD group, but was not highlighted in the comparison groups.

“*You are not able to say anything to anyone. Society isolate(s) you. No one wants to talk to you.*”.

(mother of a child with an ASD diagnosis)

“*No one will allow me in their home.*”.

(mother of a child with an ASD diagnosis)

#### 3.4.3. Life Changes, including Financial

Caregivers of a child diagnosed with ASD described financial difficulties of added costs of therapy and school. Caregivers also described life-changing decisions for the mother, such as quitting jobs, additional need to care for an autistic child, and sending other children away so they can focus their energy and resources on their autistic child. The ID comparison group demonstrated no similar mention of large life changes brought about by their child’s diagnosis.

“*I quit my job and spent time with her.*”.

(mother of a child with an ASD diagnosis)

“*Such a child costs a lot.*”.

(mother of a child with an ASD diagnosis)

#### 3.4.4. Change in Interpretation of Neurodevelopmental Disorder

Caregivers of a child diagnosed with ASD or ID described how their understanding of developmental disorders improved because of their own child’s diagnosis.

“*Now I understand it better than before… Earlier I used to think that either it will be or it won’t be. … Earlier these features [symptoms] were normal for me*.”.

(mother of a child with an ASD diagnosis)

### 3.5. Family Support

A final theme identified was that of family support and the infrastructure of that support, i.e., perceived support from partners and extended family members/community. The identified sub-themes included: (1) impact on relationships between partner and families; (2) and positive support.

#### 3.5.1. Impact on Relationships between Partner and Families

Only mothers in the ASD group reported a perceived lack of family support (including from partners), and feeling unsupported. Comparatively, mothers in the ID comparison group described greater levels of perceived acceptance of developmental disabilities by family members, and also minimal impact on relationships between family members.

*“Everyone loves her.”*.

(mother of a child with an ID diagnosis)

*“No one discriminates.”*.

(mother of a child with an ID diagnosis)

Caregivers in the ASD group reported a lack of support from their extended family, community and acceptance of their child, and associated stigma with the diagnosis, and the comparison groups made some reference to these difficulties.

“*I had no support system from the family… I mean no one accepts, even your brothers and sisters.*”.

(mother of a child with an ASD diagnosis)

“*In family.... lack acceptance. They didn’t accept. Even when I told my mother.*”.

(mother of a child with an ASD diagnosis)

#### 3.5.2. Positive Support

Some caregivers in the ASD group described the importance of being able to talk to others, and this contrasted to their feeling of being unable to do so, as outlined in sections above regarding a lack of support. This group also spoke about the importance of the support they received from their autistic child.

“*Someone who loves me truly is (child’s name) and at present I am very happy.*”.

(mother of a child with an ASD diagnosis)

## 4. Discussion

In this study, we investigated caregivers’ experiences of autism, and utilised a previously described conceptual framework by de Leeuw and colleagues [9], to explore the contextual and cultural factors impacting the recognition, interpretation and reporting of autism in an urban setting in Delhi, India. We compared caregivers’ perspectives of autism and neurodevelopmental disorders from groups of mothers with children with a diagnosis of ASD to two comparison groups: (1) parents of children diagnosed with a different developmental disorder, specifically ID; and (2) parents of children meeting their developmental milestones. We also explored the impact of a developmental disorder diagnosis across these groups. We found considerable differences between groups, especially relating to the interpretation of symptoms and the perceived impact of a diagnosis.

A striking result was the limited level of awareness of childhood developmental milestones, developmental disorders and autism across all caregivers. Limited societal awareness of developmental disorders and autism has been found in previous studies in India, including amongst health professionals [3,26]. Limited child development literacy around autism and broader issues regarding child development can impact the timing and recognition of autism symptoms [9]. A recent study, conducted only with parents of autistic children recruited from tertiary hospitals in Delhi, also found a limited awareness of autism [11]. This has similarly been reported in other LMICs in South Asia, such as families in other areas of India [3], Pakistan [27] and Nepal [4]. Our study, therefore, further emphasises the need to improve awareness around child development, developmental milestones and developmental disorders, such as autism [3,4,9,11]. Increasing awareness about autism is particularly important as it has been associated with reduced stigmatising attitudes [28,29]. Building on the results of our study, future research could pilot awareness building and changing perceptions, followed by studying how this increased awareness impacts knowledge, attitudes and beliefs in the community.

Our results demonstrated that mothers of children with an ASD diagnosis felt considerable stigma, but interestingly this did not appear to be mirrored by the experiences of caregivers in the ID group (see limitations section). High levels of stigma experienced by parents of autistic children has previously been reported in other regions of India [13,27,30,31]. Stigma can be distinguished between felt stigma (i.e., internal stigma rooted in fear of enacted stigma), and enacted stigma (external stigma, including overt ostracism or discrimination) [32]. Both can be experienced by the parents and family members of an autistic child, and this is referred to as affiliate stigma [33,34]. Affiliate stigma was strongly perceived in our interviews with the caregivers of children with a diagnosis of ASD. Caregivers reported not wanting to take their child outside, or to their extended family home, which for some led to extreme feelings of maternal isolation. A prior study conducted in Eastern India also highlighted that parents often internalize stigma, leading to associated psychological distress, which can have a negative impact on help-seeking [30]. Our results, in keeping with prior studies conducted in India, highlight the importance of reducing stigma experienced by families. One method of reducing stigma within communities may be to provide autism training to community health workers: some studies have found that autism training can improve knowledge and decrease stigmatising attitudes [9,29,35]. For example, one study conducted in Ethiopia evaluated the impact of video-based training on developmental disorders and mental health for rural community health workers. This study demonstrated a decrease in negative beliefs and stigmatising attitudes among trained health workers [35]. Similar results showing a reduction in stigmatising attitudes associated with autism training has been found in studies with students in Lebanon and the US [9,29].

The effect of stigma can be far-reaching, and it has been suggested that this experience of stigma can lead caregivers to find alternative explanations for their child’s behaviours [9]. Our results demonstrated a difference between groups in parental interpretation and explanation (explanatory models) of their child’s autism symptoms. Explanatory models describe an individual’s perspectives on the nature, causes, and course of an atypicality, illness or developmental delay [36]. These models are impacted by culture and context [9]. Interestingly, for the comparison groups, mothers’ explanations mainly focused on biological explanations; whereas, for the ASD group, mothers tended to focus on social explanations. This specifically included a lack of optimal environment in the family, stress during pregnancy, and self-blame from mothers for a lack of attention they had given to their child. As the biological causes of autism are complex and not fully understood, this uncertainty and absence may drive caregivers to develop their own models or social explanations of autism to understand and cope with the impact of a diagnosis [37]. Previous reports have found that caregivers’ explanatory models for autism might include biological models, including complications during pregnancy or birth or malnutrition [4,31,38], which were not expressed in this study. However, cultural and contextual factors may impact the popularity of these explanations [9], for example, the belief (despite much evidence to the contrary) of the impact of vaccines on autism is prevalent in North America, and also within Somali communities living in the USA and UK [39], but much less so in sub-Saharan Africa [40], and were not expressed in our interviews with caregivers in India. Supernatural explanations are also common in some contexts and have been found to be more prevalent within rural areas and LMICs [9]. Our results did not highlight such explanations, perhaps due to the urban sample interviewed. However, interviews did highlight a sense of maternal feeling of guilt or blame about their child’s autism symptoms, which was not found in the comparison groups. Similar descriptions of maternal feelings of guilt and atypical development being attributed to poor parenting and spoiling the child have been found in Nepal [4]. A study conducted in China with parents of autistic children found that affiliate stigma partially mediated the links between self-esteem and shame in families [41]. Future studies could further investigate stigma-related mediation of guilt, blame and self-esteem within the Indian context related to parental experiences of autism.

The impact of an ASD diagnosis was perceived as a profound change in life, attitude, mental health and financial stability in the ASD group. The ASD group specifically highlighted social/parental isolation, feelings of depression, concern for the future, disbelief about the diagnosis, lack of support/acceptance in the family, as well as big life changes (e.g., mother leaving job). These results from the ASD group confirm previous findings (e.g., [42]), and specifically, a meta-synthesis of studies investigating parental experience of autism around the world identified a common theme of feeling socially excluded and negatively judged by others, even within their own extended family [43]. Financial difficulties and concerns for the child’s future have also been highlighted as two of the six main factors associated with parenting stress for parents of children with ASD in South East Asia in a systematic review [2]. However, interestingly, these were not identified by the comparison groups as a potential impact of an ASD diagnosis for families. It is possible that this is reflective of the small sample size (see limitations), but it is also possible that such stressors within an urban environment may be more associated with the uncertainty of the causes and impact of autism, and the limited awareness of autism compared to more “overt” disabilities that may be experienced externally by children with ID, as well as more established health services in India for disability, than for autism.

### Limitations

The strengths of this paper lie in the inclusion of caregivers of children, both with and without a diagnosis of ASD in an under-researched population in urban Delhi in India. However, comparison group families were often unaware of the term “autism”, and this may impact their consideration of how a diagnosis might impact a family. Despite this, we consider it important to understand the perceptions of such caregivers, in order to gain an insight into general community perceptions of what families are experiencing and their accompanying stigma. There are two additional limitations of this work. First, our caregiver groups were not matched in terms of socioeconomic indicators, e.g., maternal education. The mothers in the ASD group generally had a higher level of education (tertiary education) than in the other two groups. This may impact our conclusions, as higher levels of education have been associated with higher levels of awareness of healthcare and autism traits. In turn, this may therefore be associated with being able to successfully start and navigate the pathway to autism diagnosis. However, this difference was also potentially illustrative of the demographics of parents able to navigate the health system pathways to obtain an ASD diagnosis for their child [11]. The second key limitation of this study is the small sample size across the groups. However, despite this, we felt that we reached saturation in our responses, and previous methodological papers have justified small sample sizes of as few as six interviews upon reaching saturation [44].

## 5. Conclusions

Our results highlight the acute feelings of social isolation and lack of support experienced by mothers of a child diagnosed with ASD in the communities we were working with in Delhi. This study demonstrates a vital need for greater community awareness and recognition of autism in India, including in urban regions such as Delhi. This may help reduce stigma within communities, and discrimination from service providers, as well as facilitate wider family support, for example, through autism and mental health training of community health workers. There is a need for increased research to understand autism from multiple perspectives, which can help increase the engagement of communities in evidence-based health services, leading to strengthened health systems, and ultimately greater support being provided to children and their families.

## Figures and Tables

**Table 1:** Participant demographics.

Demographics			Autism Group (*n* = 5)	Comparison Groups	
				Child Meeting Their Developmental Milestones (*n* = 4)	Child with Neurodevelopmental Condition (ID) (*n* = 4)
Child characteristics	Child age (years)	Median (range)	3.89 (3.57–6.18)	4.97 (3.05–6.30)	6.54 (3.47–6.71)
	Sex	No. Female (Male)	3 (2)	3 (1)	2 (2)
	Child development (DP-3, general developmental score)	Median (range)	53 (<40–71)	98.5 (91–15)	<40 (<40–62)
	ASD traits (INDT total score)	Median (range)	6 (4–9)	0 (0)	1 (0–2)
	Social Communication Quotient score	Median (range)	6 (5–16)	2.5 (2–6)	8.5 (3–14)
Family Resources	Mother’s education	Secondary	1	3	3
		Tertiary	4	1	1
	Father’s education	Secondary	0	4	1
		Tertiary	5	0	3
	Mother’s age (years)	Median (range)	35 (28–36) *	28 (27–29) *	29.5 (25–38)
	Mother’s occupation	Not working	3	1	4
		Professional or skilled job	2	1	0
		Unskilled job	0	2	0
	Father’s occupation	Not working	0	0	0
		Professional or skilled job	4	3	4
		Unskilled job	1	1	0

* Missing data for one participant.

## Data Availability

Due to the nature of this research, participants of this study did not agree to their data being shared publicly, so supporting data is not available.
